# Regulation of distal tubule sodium transport: mechanisms and roles in homeostasis and pathophysiology

**DOI:** 10.1007/s00424-022-02732-5

**Published:** 2022-07-27

**Authors:** David Pearce, Anna D. Manis, Viatcheslav Nesterov, Christoph Korbmacher

**Affiliations:** 1 Department of Medicine, Division of Nephrology, and Department of Cellular and Molecular Pharmacology, University of California San Francisco, San Francisco, CA USA; 2 Institut für Zelluläre und Molekulare Physiologie, Friedrich-Alexander-Universität Erlangen-Nürnberg, Erlangen, Germany, Erlangen, Germany

**Keywords:** Na^+^-Cl^−^ cotransporter (NCC), Epithelial sodium channel (ENaC), Renal outer medullary K^+^ channel (ROMK), Aldosterone, Mineralocorticoid receptor (MR), 11ß-hydroxysteroid dehydrogenase type 2 (11ßHSD2), Serum and glucocorticoid-regulated kinase 1 (SGK1); mTOR complex 2 (mTORC2), With no lysine, Kinase 1 and 4 (WNK1 and WNK4), Aldosterone-sensitive distal nephron (ASDN), Early distal convoluted tubule (DCT1), Late distal convoluted tubule (DCT2), Connecting tubule (CNT), Cortical collecting duct (CCD)

## Abstract

Regulated Na^+^ transport in the distal nephron is of fundamental importance to fluid and electrolyte homeostasis. Further upstream, Na^+^ is the principal driver of secondary active transport of numerous organic and inorganic solutes. In the distal nephron, Na^+^ continues to play a central role in controlling the body levels and concentrations of a more select group of ions, including K^+^, Ca^++^, Mg^++^, Cl^−^, and HCO_3_^−^, as well as water. Also, of paramount importance are transport mechanisms aimed at controlling the total level of Na^+^ itself in the body, as well as its concentrations in intracellular and extracellular compartments. Over the last several decades, the transporters involved in moving Na^+^ in the distal nephron, and directly or indirectly coupling its movement to that of other ions have been identified, and their interrelationships brought into focus. Just as importantly, the signaling systems and their components—kinases, ubiquitin ligases, phosphatases, transcription factors, and others—have also been identified and many of their actions elucidated. This review will touch on selected aspects of ion transport regulation, and its impact on fluid and electrolyte homeostasis. A particular focus will be on emerging evidence for site-specific regulation of the epithelial sodium channel (ENaC) and its role in both Na^+^ and K^+^ homeostasis. In this context, the critical regulatory roles of aldosterone, the mineralocorticoid receptor (MR), and the kinases SGK1 and mTORC2 will be highlighted. This includes a discussion of the newly established concept that local K^+^ concentrations are involved in the reciprocal regulation of Na^+^-Cl^−^ cotransporter (NCC) and ENaC activity to adjust renal K^+^ secretion to dietary intake.

## Introduction

**Fig. 1:**
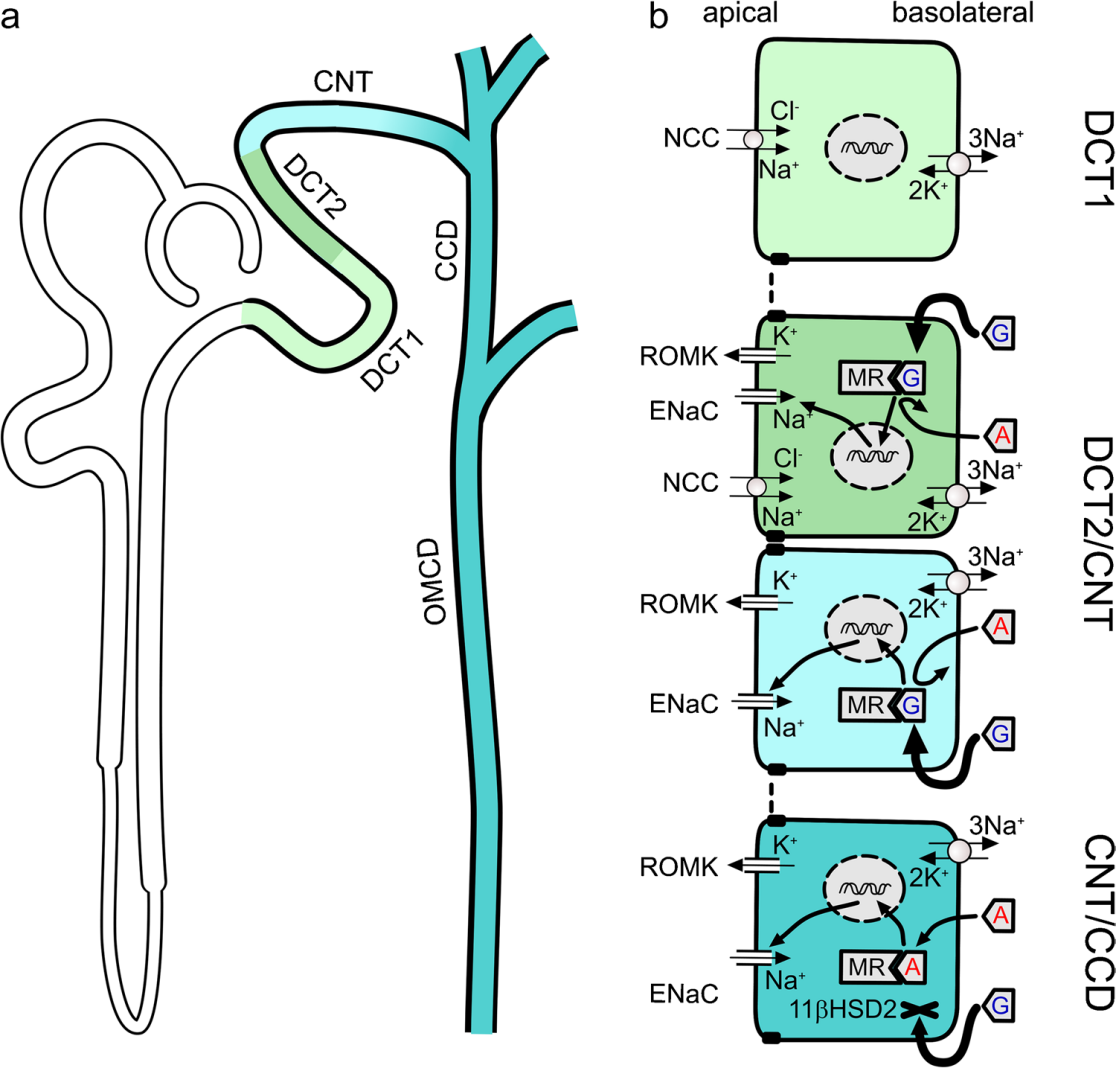
Segment-specific sodium transport mechanisms in the distal nephron. **a** Schematic representation of a single nephron highlighting different segments of the distal nephron, i.e., the distal convoluted tubule with its early (DCT1) and late (DCT2) portion, the connecting tubule (CNT), the cortical collecting duct (CCD), and the outer medullary collecting duct (OMCD). **b** Tubule epithelial cell models illustrating segment-specific apical sodium uptake mechanisms. Basolateral sodium extrusion in exchange for potassium (3Na^+^/2 K^+^) is accomplished by the basolateral Na^+^-K^+^-ATPase in all cell types. A defining feature of both DCT1 and DCT2 is the apical Na^+^-Cl^−^ cotransporter (NCC); DCT2, but not DCT1, also expresses the epithelial sodium channel (ENaC). ENaC is the sole apical sodium uptake mechanism in CNT and CCD principal cells. In addition to playing a decisive role in fine tuning renal sodium absorption, ENaC also generates the electrical driving force necessary for K^+^ secretion meditated primarily by the apical renal outer medullary K^+^ channel (ROMK). In the late CNT and entire CCD (CNT/CCD), aldosterone (A) is the key hormonal activator of ENaC through the mineralocorticoid receptor (MR) which is protected from glucocorticoid action by 11ß-hydroxysteroid dehydrogenase type 2 (11βHSD2). In the DCT2 and early CNT (DCT2/CNT), MR appears to have constitutive activity, possibly due to low levels of 11βHSD2, allowing glucocorticoids (G) to activate the receptor. This provides a potential explanation for the aldosterone-independent but MR-dependent ENaC activity in the latter region, which is probably important for Na^+^ homeostasis and blood pressure control, as well as aldosterone-independent K^+^ secretion

Sodium is central to human biology in large measure due to its essential roles in establishing electrochemical gradients across the plasma membrane of virtually all cells, and in controlling the absorption, distribution, and excretion of water and a multitude of electrolytes and organic solutes. Consequently, sodium ion (Na^+^) transport is tightly regulated at all points of compartment separation. This review will focus on Na^+^ transport in the cortical distal nephron, comprising the distal convoluted tubule (DCT), connecting tubule (CNT), and cortical collecting duct (CCD), shown schematically in Fig. 1. Here, transepithelial ion transport and water reabsorption are fine tuned to adapt to changes in electrolyte concentrations and the overall fluid and electrolyte content of the body. In this context, Na^+^ transport in the distal nephron not only plays a central role in determining the body’s overall Na^+^ content therefore blood pressure, but it also critically impacts the excretion of other ions and is central to regulating extracellular fluid (ECF) electrolyte concentrations. This additional regulatory role of distal tubular Na^+^ transport is strikingly displayed in the control of ECF K^+^ concentration [K^+^]. It is well known that Na^+^ mishandling can cause both hyperkalemia and hypokalemia, and their reciprocal effects on each other’s transport are increasingly recognized [62, 68, 97, 161]. Other articles in this collection will go into detail regarding the transport and regulation of K^+^ [95], and blood pressure [173]. Here, we will particularly focus on two Na^+^ transport mediators and their regulatory interplay: in the DCT, the electroneutral Na^+^-Cl^−^ cotransporter (NCC) and in the aldosterone-sensitive distal nephron (ASDN), the epithelial Na^+^ channel (ENaC) (Fig. 1). We will primarily address key aspects of their regulation, with special attention to the steroid hormone, aldosterone, and local effects of peritubular K^+^. In addition to their roles in controlling overall body Na^+^ content, and hence extracellular fluid volume and blood pressure, the relative activities of these two apical membrane mediators of Na^+^ entry play key roles in establishing luminal conditions that favor or disfavor K^+^ secretion. Water transport, which is central to all aspects of fluid and electrolyte homeostasis [41], will not be addressed here.

## Aldosterone regulation of Na^+^ transport in the distal nephron

Aldosterone is the key hormonal factor regulating Na^+^ transport in the distal nephron, and for coupling Na^+^ reabsorption with K^+^ secretion. Aldosterone was discovered as “the mineralocorticoid” factor in beef adrenal extracts as assessed by a bio-assay based on the effect of corticosteroid fractions on the urinary Na^+^/K^+^ ratio in adrenalectomized rats [165]. Thus, from the very inception of aldosterone research, its role in concomitantly controlling both Na^+^ and K^+^ transport was its defining characteristic. Decades of endocrine signaling, ion transport, and electrolyte research have led to a cohesive picture of aldosterone action and pathophysiologic roles, which have been elaborated in other reviews [82, 86, 138], including in this collection [173]. For the present discussion of Na^+^ transport, we will emphasize direct and indirect effects on ENaC and NCC.

Although preceded by a latent period, the initial effects of aldosterone are quite rapid for a genomic mechanism [174]: changes in Na^+^/K^+^ ratio occur within 30–60’ in dogs injected with aldosterone into their renal artery [52], and in 1–2 h in cultured cells [12, 51, 63]. These effects are mediated by the mineralocorticoid receptor (MR), which directly regulates transcription of a large group of genes that impact Na^+^ transport. These include SGK1, αENaC itself, Dot1, FKBP5, and GILZ [31, 88, 100, 119, 154, 162, 172, 196]. ENaC is highly expressed in the apical membrane of principal cells (PCs) of the ASDN and mediates Na^+^ entry from lumen to cell. The electrochemical gradient that drives apical Na^+^ entry and ultimately Na^+^ translocation is established by the basolateral Na, K-ATPase, the activity of which must be coordinated with that of ENaC [40]. Although the ASDN re-absorbs less than 10% of the filtered Na^+^ load, it is critical for modulating the amount of Na^+^ that appears in the urine in response to changes in intake [84, 92, 112].

It is important to note that although aldosterone acts almost exclusively through MR, MR is not similarly exclusive in its response: The major glucocorticoids (cortisol in primates, corticosterone in rodents) bind with high affinity and potently activate MR [80, 147]. MR is guarded from glucocorticoid activation in some tissues by the enzyme 11ß-hydroxysteroid dehydrogenase type 2 (11ßHSD2), which converts both cortisol and corticosterone, but not aldosterone, to steroids with very little affinity for MR or glucocorticoid receptor (GR) [50]. 11ßHSD2 is highly expressed in some—but not all—parts of the distal nephron, which has been recently found to be of significance for aldosterone-independent control of ion transport and in particular regulation of K^+^ homeostasis [90, 104, 106, 171, 185], as is further expanded below.

## The epithelial sodium channel in the aldosterone-sensitive distal nephron

The ASDN consists of the second part of the distal convoluted tubule (DCT2), the connecting tubule (CNT), and the collecting duct (CD) with its various portions, i.e., the cortical collecting duct (CCD) and outer and inner medullary collecting duct (MCD). In the absence of aldosterone, MCD ENaC activity is much lower than in that of cortical ASDN, but it is markedly stimulated by aldosterone, despite having slightly lower MR expression than CNT and CCD [45, 170]. In the ASDN, ENaC and the renal outer medullary K^+^ channel (ROMK; also known as Kir1.1 or KCNJ1) are rate-limiting steps for transepithelial sodium absorption and potassium secretion, respectively [137, 180]. Both channels are localized in the apical cell membrane of principal cells in the CNT and CD and in principal-like cells in the DCT2 [38, 85, 105, 192]. ENaC belongs to the ENaC/DEG family of ion channels, and comprehensive recent reviews exist about its physiology and pathophysiology, its structure and function, and its highly complex regulation [77, 102, 144]. As addressed in more detail in another contribution to this issue [95], additional apical potassium channels may contribute to potassium secretion under certain physiological conditions, e.g., the small conductance calcium-activated potassium channel 3 (SK3) [15] and Maxi-K (BK) channels [124], but ROMK is thought to be the major potassium secretory pathway [180]. In the present context, it is important to note the critical role that ENaC plays in controlling the driving force for K^+^ secretion. The functional importance of ENaC in this context, and in controlling volume and blood pressure, is highlighted by gain-of-function mutations of the channel causing Liddle syndrome, and loss-of-function mutations causing pseudohypoaldosteronism (PHA-1) [139]. PHA-I-inducing loss-of-function mutations of ENaC are characterized by renal salt wasting and hyperkalemia [141]. In contrast, Liddle syndrome is a severe form of salt-sensitive arterial hypertension associated with hypokalemia [141]. The hypokalemia seen in Liddle syndrome and hyperkalemia seen in PHA-I are strong reminders of the importance of ENaC in K^+^ excretion [139]. Liddle syndrome is rare [21, 83] but provides molecular proof that an inappropriate increase in ENaC activity results in hypertension. Thus, subtle disturbances of ENaC regulation resulting in increased ENaC activity are likely to contribute to the pathophysiology of essential hypertension, particularly in a subset of patients with salt-sensitive hypertension [61, 102]. Indeed, many drugs used to treat essential hypertension ultimately decrease renal ENaC activity, either indirectly by reducing the activity of the renin–angiotensin–aldosterone system (RAAS) or more directly by inhibiting the mineralocorticoid receptor (MR) or the channel itself. MR antagonists, like spironolactone, finerenone [131], and eplerenone, and ENaC inhibitors, like amiloride and triamterene, have a blood pressure lowering effect [145], particularly in patients with resistant hypertension [181, 182]. The majority of mutations causing Liddle syndrome disrupt PY motifs localized in the C termini of the β- or γ-subunit of ENaC [140]. These PY motifs serve as binding sites for the ubiquitin ligase, NEDD4-2, a NEDD4 (neural precursor cell–expressed developmentally downregulated protein 4) family member [34, 158]. Binding at these sites facilitates NEDD4-2-mediated channel ubiquitination with subsequent channel retrieval and proteasomal degradation [143]. Mutations in the PY motifs disrupt the physiological NEDD4-2/ENaC interaction. This results in an increase in the number of channels at the cell surface, which is thought to be the main mechanism by which Liddle syndrome mutations cause a gain-of-function effect [75]. In addition, these mutations have been reported to reduce Na^+^ feedback inhibition [76] and to increase the channel’s responsiveness to aldosterone [10, 16, 33]. The underlying mechanism for this enhanced aldosterone sensitivity is unclear but may involve increased trafficking of ENaC to the cell membrane. Moreover, mutations affecting the PY motif of β-ENaC have been reported to increase channel open probability [5] possibly due to enhanced proteolytic channel activation [78]. The latter mechanism is a unique feature of ENaC [142] and involves cleavage at specific sites in its α- and γ-subunit, resulting in the release of inhibitory tracts. However, the complex mechanisms contributing to proteolytic ENaC activation are still not fully understood, and physiologically relevant proteases remain to be identified [4, 77].

Prostasin (PRSS8) was the first membrane-anchored serine protease demonstrated to activate ENaC in coexpression experiments and was therefore named channel activating protease 1 (CAP1). However, it remains an open question whether PRSS8 contributes to proteolytic ENaC regulation in the kidney in vivo [37, 39]. Recently, transmembrane serine protease 2 (TMPRSS2 or epitheliasin), which is highly expressed in several epithelial tissues including renal distal tubule, has been identified as a likely candidate to contribute to proteolytic ENaC activation [163]. Inappropriate proteolytic ENaC activation by urinary plasmin may contribute to sodium retention and edema formation in nephrotic syndrome [19, 30, 116, 164], but the pathophysiological role of plasmin remains a matter of debate [18, 66], and additional aberrantly filtered urinary proteases are likely to be involved [2, 8, 9, 65].

Interestingly, several gain-of-function mutations have been identified also in the extracellular regions of ENaC subunits [28, 77, 130]. They are thought to cause Liddle syndrome in affected patients [149], but may also contribute to the pathophysiology of atypical cystic fibrosis without mutations in CFTR [128, 129]. These mutations primarily affect channel gating resulting in increased channel open probability. Thus, ENaC regulation is highly complex, and channel activity may be affected at different levels under pathophysiological conditions [144]. Recently, the first cryo-EM structures of ENaC became available [108, 109]. These structural data open up exciting new horizons to study ENaC function at the molecular level [77, 195].

### Consequences of ENaC activation and functional interdependence of sodium absorption and potassium secretion in the ASDN

Increased ENaC-mediated Na^+^ absorption depolarizes the apical membrane which enhances the driving force for K^+^ secretion via ROMK and BK channels [179]. Importantly, this electrogenic Na^+^ transport is augmented by Na^+^ delivery, which is enhanced by inhibition of upstream transporters; sodium-chloride cotransporter (NCC) and Na–K-2Cl cotransporter 2 (NKCC2) have received the bulk of recent attention [62, 67], but data support a role for sodium-hydrogen exchanger 3 (NHE3), as well [191]. One central mechanism for enhancing distal Na^+^ delivery is inhibition of the thiazide-sensitive NCC through a WNK (with no lysine kinase)/SPAK (SPS1-related proline/alanine-rich kinase)–dependent mechanism [166, 26]. The resulting increase in distal sodium delivery—in the presence of adequate active ENaC—stimulates the driving force for ROMK-mediated potassium secretion. Importantly, however, increased Na^+^ delivery due to NCC inhibition in-and-of-itself does not stimulate K^+^ secretion. In particular, Hunter et al. showed that acute NCC inhibition using hydrocholorthiazide does not trigger a kaliuresis [70], and Ayasse et al. found that the effect of furosemide to induce a kaliuresis depends on ENaC expression [11]. In contrast, K^+^ administration rapidly stimulates ENaC activity concomitantly with NCC inhibition, even prior to a significant rise in aldosterone, an effect which is only partially inhibited by MR blockade with eplerenone [161]. Interestingly, cell culture experiments strongly support the idea that these effects of K^+^ on ENaC activity are direct and mediated at least in part by cell autonomous activation of the mTORC2-SGK1 signaling module in PCs [161]. Another potential contributing factor is that increased tubular flow is believed to directly activate ENaC through effects on open probability [101], as well as apical BK channels possibly via TRPV4 (transient receptor potential vanilloid 4 cation channel)–mediated calcium inflow [180]. Clearly, a high-K^+^ diet increases ROMK activity, which plays an important role in long-term stimulation of potassium secretion [177]. An increase in apical potassium conductance not only enhances potassium secretion but also favors ENaC-mediated sodium absorption due to the hyperpolarization of the apical membrane potential, thus highlighting the complex functional interdependence of ENaC and ROMK in the ASDN.

A conundrum is raised by the importance of aldosterone in responding to both volume depletion and hyperkalemia: How does the ASDN “know” to respond to aldosterone with an increase in NaCl reabsorption vs. increased K^+^ secretion? Several—non mutually exclusive—theories have been proposed for this “aldosterone paradox,” which remains not fully understood [7, 136]. Regulation of upstream electroneutral Na^+^ reabsorption may contribute to the regulation of electrogenic Na^+^ reabsorption [97]; however, it is not sufficient [70], and other factors must obtain. Numerous hormonal and local factors that regulate ENaC and ROMK may be implicated [54, 84, 137, 180]. Angiotensin II is a logical candidate for shifting toward NaCl reabsorption since it rises in response to volume depletion or lowered blood pressure. Two mechanisms warrant note: (1) Angiotensin II–induced dephosphorylation of MR in its hormone binding domain (MR/S843) in intercalated cells [157]. When phosphorylated at this residue, MR cannot bind aldosterone; hence, the dephosphorylation is permissive for MR activation. Cl^−^ absorption through intercalated cells is activated and together with ENaC activation in principal cells increases NaCl reabsorption. Hyperkalemia has the opposite effect, leading to phosphorylation of MR/S843, and inactivation of intercalated cell MR. (2) Angiotensin II–induced modulation of WNK4-kelch-like 3 signaling favors NCC activation [24]. K^+^ has the opposite effect. WNK kinase regulation of NCC is discussed further below. As discussed below, K^+^ itself also has direct effects mediated by mTORC2 possibly in collaboration with WNK1 and/or WNK4, which alter the net consequences of aldosterone signaling.

## The aldosterone-MR-SGK1 signaling module regulates ENaC

SGK1 (serum and glucocorticoid-regulated kinase 1) was first identified as a glucocorticoid-regulated gene in a breast cancer cell line. Its role in mediating effects of aldosterone, however, was recognized later when it was independently cloned from collecting duct cell lines as an aldosterone-regulated gene, and shown to regulate ENaC [31, 103]. SGK1 gene transcription is rapidly stimulated by corticosteroids (aldosterone or cortisol/corticosterone), responding in less than 15’ in cultured cells and within 30’ in animals [17, 23, 31, 103], with protein levels rising shortly thereafter. A variety of mechanisms and targets for SGK1 have been suggested, some of which play direct roles in regulating electrolyte homeostasis [196], others of which likely act indirectly. For example, ENaC retrieval from the plasma membrane and degradation are regulated, at least in part by SGK1 phosphorylation and inhibition of the ubiquitin ligase, Nedd4-2 [34, 159]. This led to the concept that aldosterone increases ENaC surface expression through diminished ubiquitination and inhibited internalization, which is well supported by data from expression systems. However, data have been mixed in in vivo experiments [44, 133]. Although the mechanism is less well understood, SGK1 also stimulates trafficking to the membrane [3, 118, 127]. Additionally, a smaller but more rapid effect is elicited by direct phosphorylation of the channel’s α-subunit to increase its open probability [35, 169]. Recent evidence suggests that phosphorylation of this stimulatory site in the C-terminus of α-ENaC may not necessarily be accomplished by SGK1 itself but may be mediated by the dual-specificity tyrosine phosphorylated and regulated kinase 2 (DYRK2) [36]. Moreover, phosphorylation of this site may prime a highly conserved preceding serine residue to be phosphorylated by glycogen synthase kinase 3 β (GSK3β), resulting in channel inhibition which may limit the initial stimulatory effect and serve as feedback inhibition. Interestingly, SGK1 is known to inactivate GSK3β. Thus, SGK1 induced by aldosterone may activate ENaC also in part by inactivation of GSK3β [148].

SGK1 regulation of a variety of channels and transporters other than ENaC has been suggested with varying degrees of certainty, including TRPV5, ROMK, KCNE1/KCNQ1, ClC-Kb, NHE3, NKCC2, NCC, and SGLT1, and the Na^+^/K^+^-ATPase as reviewed in ref. [81]. In the present context, it is notable that recent evidence strongly supports the conclusion that effects of aldosterone and SGK1 on NCC are indirect and due to ENaC-dependent lowering of plasma [K^+^], which stimulates NCC [32, 79, 166].

It is also important to note that MR-regulated genes other than SGK1 are clearly physiologically critical, as witnessed by the dramatic differences between loss of MR vs. SGK1 function in animal studies [13, 14, 132, 186]. The full spectrum of such genes remains unknown. In particular, SGK2 and 3, close relatives of SGK1 with similar substrate specificity, are not aldosterone regulated, and findings regarding their effects on tubule ion transporters and channels have been mixed [43, 64, 114, 115].

### mTORC2-dependent activation of SGK1

**Fig. 2:**
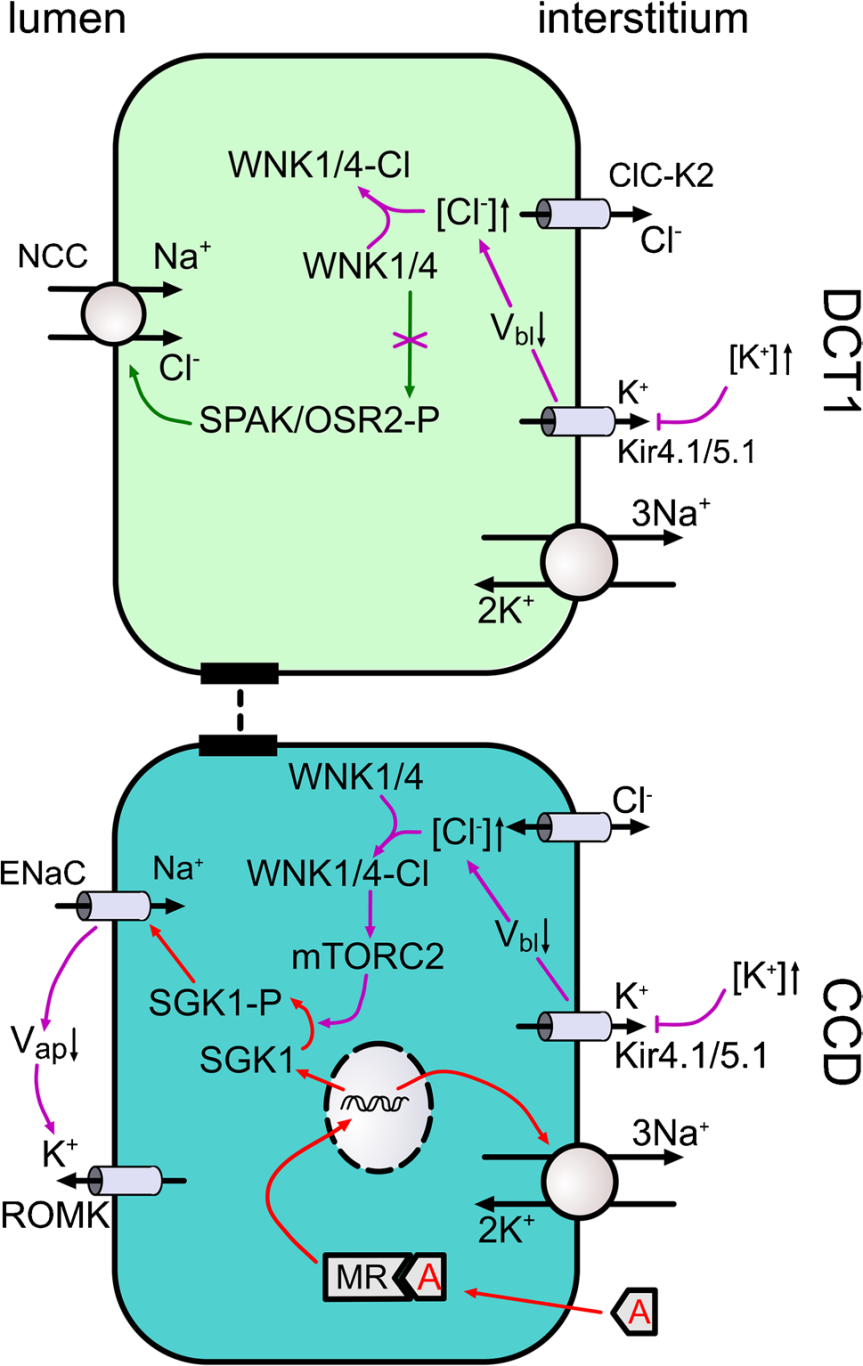
Coordinated regulation of ENaC and NCC by interstitial potassium. The effects of increased interstitial K^+^ on Na^+^ transport are shown for a DCT1 cell (top) and CNT/CCD cell (bottom). Baseline membrane potential is controlled primarily by Kir4.1/5.1. Increased interstitial K^+^ concentration ([K^+^]↑) depolarizes the basolateral membrane potential (V_bl_↓), thus altering the electrochemical gradient for Cl^−^ across the basolateral membrane equipped with Cl^−^ channels (in particular ClC-K2 in DCT1), and eventually causes an increase in intracellular Cl^−^ concentration ([Cl^−^]↑) in both the DCT1 and CCD. Chloride can then bind to WNK1/4, which inhibits its kinase activity and prevents NCC activation in the DCT1. In the CCD, chloride-bound WNK1/4 interacts with both mTORC2 and SGK1 to increase SGK1 phosphorylation and subsequent ENaC activation. Increased electrogenic ENaC activity depolarizes the apical membrane potential (V_ap_↓), thereby stimulating ROMK-mediated K^+^ secretion. Aldosterone (A) contributes to ENaC regulation in the CCD by binding to the mineralocorticoid receptor (MR) and increasing SGK1 transcription. Purple arrows indicate effects due to an increase in interstitial K.^+^ and red arrows depict the effects of aldosterone (A)

One of the most striking features of SGK1 is that it is under dual regulation: its expression level by direct aldosterone/MR-stimulation of gene transcription (Fig. 2), and its activity through mTORC2-dependent phosphorylation of its C-terminal hydrophobic motif (HM) [53, 89]. HM phosphorylation was originally identified for SGK1’s cousins, Akt and PKC [74, 152], and subsequently for SGK1 [53], and its role in ENaC regulation was demonstrated in cultured cells [89] and in vivo [56]. mTORC2 is a multi-protein complex comprising the serine-threonine kinase mTOR and three accessory proteins—mLST8, Rictor, and mSin1—which control multiple aspects of substrate specificity [49]. Interestingly, both insulin and angiotensin II stimulate mTORC2-dependent activation of SGK1 and ENaC [57], which may play a role in the pathogenesis of salt-sensitive hypertension found in type 2 diabetes and the metabolic syndrome [82]. However, the central physiological role of mTORC2 appears to be regulation of K^+^ secretion. Initial in vivo support for this came with characterization of renal tubule–specific Rictor knockout mice, which have low levels of phosphorylated SGK1, elevated aldosterone, and decreased ROMK but normal ENaC activity on a normal Na^+^/normal K^+^ diet [60]. Interestingly, the mice tolerate Na^+^ restriction well, however exhibit striking hyperkalemia with severe natriuresis and decreased GFR on a prolonged high-K^+^ diet. Hyperkalemia became lethal when animals were treated with the ENaC inhibitor triamterene suggesting a more profound defect in ROMK than in ENaC [60]. It is notable that in contrast with Rictor KO mice [60], either acute treatment of mice with an mTOR inhibitor [56] or mTOR gene deletion [27] markedly reduces ENaC activity. Although these apparently conflicting findings require additional study to reconcile, they are consistent with the possibility that in vivo mTORC1 and mTORC2 are *both* able to stimulate ENaC (via SGK1), while only mTORC2 regulates ROMK. It should be reiterated that mTOR has substrates other than SGK1—for example, PKC, Akt, 4EBP, or p70-S6-kinase which might be implicated in ENaC and ROMK regulation. Notably, PKC has been shown to phosphorylate and inhibit ROMK [194].

More recently, Sørensen et al. found that acute effects of a K^+^ load on K^+^ excretion (during the first 4 h following a KCl load by gavage) depend on ENaC activation and are largely aldosterone and MR-independent [161]. In cultured collecting duct cells, elevated [K^+^] stimulated ENaC by activating mTORC2 through a mechanism requiring basolateral Kir4.1/5.1 K^+^ channels. No change in aldosterone was required. The authors concluded that an acute K^+^ load stimulates ENaC activity directly in PCs through the mTORC2/SGK1 signaling module, and this effect is integrated with that of aldosterone by SGK1 [161]. Interestingly, WNK1 was essential for maximal SGK1 phosphorylation and was proposed to mediate the K^+^ effect, possibly by binding Cl^−^, as has been described in the regulation of NCC in DCT cells [168] (discussed further below). Furthermore, although principal cells do not support transcellular Cl^−^ transport, basolateral Cl^−^ channels have been detected [111, 151], and provide a potential mechanism for K^+^-induced changes in membrane potential to cause changes in intracellular [Cl^−^]. Additional support for the idea that the CNT/CD directly senses K^+^ is also provided by the observation that CNT/CD-specific KCNJ10 KO mice (lacking Kir4.1) have disturbed K^+^ homeostasis and impaired K^+^-dependent regulation of ENaC and ROMK [122].

## Regulation of NCC in the DCT: role of WNK kinases

Although both ENaC and NCC are critical determinants of sodium balance, and thereby modulate blood volume and blood pressure, their expression patterns and regulation are distinct. NCC is expressed in the apical membrane of DCT cells, and only colocalizes with ENaC in the DCT2. NCC is the therapeutic target of thiazide diuretics and alterations in NCC have been associated with hypertension and salt sensitivity. Inactivating mutations in NCC (gene name: SLC12A3) result in Gitelman’s syndrome, a salt-wasting tubulopathy with hypokalemia, hypomagnesemia, metabolic alkalosis, and often (but not always) low blood pressure resulting from impairment in Na^+^ reabsorption [55, 98]. This phenotype was partially recapitulated in mouse models; however, impairment in blood pressure control only became evident in NCC knockouts when fed a Na^+^ deficient diet [87, 153]. It has been suggested that the Cl^−^/HCO_3_^−^ exchanger pendrin, which is expressed apically in DCT2 intercalated cells, may partially compensate for the loss of NCC, potentially contributing to the inconsistent presentation of low blood pressure in Gitelman’s syndrome as well as the mild phenotype found in NCC knockout mice [117]. Furthermore, a double knockout of both NCC and pendrin resulted in much more severe volume depletion and ultimately renal failure [160]. The partially overlapping roles of ENaC, NCC, and pendrin emphasize the physiological importance of Na^+^ reabsorption in the distal nephron.

NCC is regulated by multisite phosphorylation, glycosylation, and ubiquitination, and its regulation can be heavily influenced by physiological and dietary factors. NCC has been shown to be highly regulated by dietary K^+^ intake and intracellular Cl^−^. It is now widely accepted that the predominant regulation of NCC is through WNK (with no lysine) kinases, which are characterized by and named for the atypical location of their catalytic lysine residue in subdomain I, rather II [188]. The key observations leading to this view have been well reviewed [62, 96, 120] and are only briefly addressed here. Of the WNK family of serine/threonine kinases, WNK1 and WNK4 are found in the DCT, but WNK4 is widely considered the major regulator of NCC [26]. A kidney-specific isoform of WNK1 (KS-WNK1), which lacks the kinase domain, is also expressed in the DCT. It has been reported to activate WNK4 and thus NCC, but its overall contribution to NCC regulation is still disputed [6]. WNK kinase activity is largely determined by intracellular chloride concentration and WNK4 has been shown to have higher chloride sensitivity compared to WNK1 [167]. Intracellular Cl^−^ is highly sensitive to changes in plasma [K^+^] and influenced by dietary K^+^ intake. Thus, NCC is regulated by K^+^ and Cl^−^ indirectly through WNK4. High plasma K^+^ causes depolarization of the basolateral membrane, possibly by altering the Nernst potential for K^+^ and/or inhibiting Kir4.1/5.1 channels, inhibiting Cl^−^ efflux through ClC-K2 channels, which increases intracellular [Cl^−^] [168, 178]. When Cl^−^ is present in sufficient amounts, it binds to WNK and inhibits its activation by autophosphorylation at serine 328 [123]. However, in a low chloride environment, WNK1 and WNK4 autophosphorylate and self-activate to bind, phosphorylate, and activate downstream effectors SPAK and OSR1 (oxidative stress–responsive kinase 1) [175]. SPAK and OSR1 form a complex with scaffolding protein MO25 (mouse protein-25), which significantly enhances phosphorylation and activity of NCC [42]. Phosphorylation of NCC not only increases its activity, but also decreases its ubiquitination and subsequent internalization [134, 135].

In humans, gain-of-function mutations in WNK1 and WNK4 result in Familial Hyperkalemia and Hypertension (FHHt; sometimes referred to as pseudohypoaldosteronism type II (PHAII) or Gordon Syndrome), a monogenic form of secondary hypertension characterized by hypertension and hyperkalemia [59, 93, 183]. The phenotypes associated with FHHt can be generally considered the inverse of Gitelman’s syndrome. Moreover, inhibiting NCC using thiazide diuretics is often sufficient to correct the clinical features of this disease, again emphasizing WNK as the major regulator of NCC. FHHt resulting from gain-of-function mutations in WNK4 is associated with more severe phenotypes than mutations in WNK1, suggesting WNK4 as the predominant regulator of NCC. WNK4 is directly regulated by E3 ligase mediated ubiquitination via Cullin 3 (CUL3) and its substrate adapter, Kelch-like 3 (KLHL3), thus mutations in CUL3 and KLHL3 also result in FHHt [22, 72]. Chen et al. were able to recapitulate the FHHt phenotype with a knockin of a mutant Cl^−^ insensitive WNK4, demonstrating that chloride sensitivity is requisite for WNK4 modulation of NCC [29]. Conversely, WNK4 knockout mice exhibited phenotypes analogous to Gitelman’s syndrome [25].

Dietary composition is a significant factor in NCC regulation both acutely and secondarily by triggering changes in RAAS hormones. NCC is responsive to dietary alterations, translating the Na^+^/K^+^ ratio to appropriate alterations in blood pressure. For example, NCC has been shown to be required for the antihypertensive effects of a high-K^+^ diet [168, 176]. Aldosterone and angiotensin II both regulate NCC activity; however, through distinct mechanisms. Angiotensin II has been reported to increase membrane expression of NCC [150], possibly through effects on WNK4 and KLHL3 [156]. Acute stimulation by aldosterone is now thought to be indirect and mediated predominantly by changes in local K^+^ concentration [32, 166], which most likely occur due to aldosterone stimulation of ENaC and possibly ROMK [32, 73, 166, 184]. An increase in ECF [K^+^] has the opposite effect, inhibiting WNK/SPAK-dependent NCC activation [121]. KLHL3 and CUL3 are required for this K^+^-dependent modulation of NCC [155].

## Site-specific regulation of ENaC and ROMK in the distal nephron

At present, perspectives regarding the functional interplay of ENaC and ROMK in the context of dietary K^+^ challenges and their mechanisms of regulation at the systemic and molecular level are undergoing a shift. In the ASDN, the homeostatic roles of DCT2 and CNT have probably been underestimated, whereas the role of the CCD may have been overemphasized [84, 99]. Recent evidence has increasingly supported the idea that in the ASDN, ENaC and ROMK are expressed and regulated in a site-specific manner which adds to the complexity of their functional interplay [90, 104–107, 171, 185, 192]. ENaC-mediated electrogenic Na^+^ absorption must be accompanied either by parallel Cl^−^ absorption, which is probably paracellular in the ASDN [58, 69, 193], or by K^+^ secretion predominantly via ROMK. Consequently, high ENaC activity in the DCT2/CNT can contribute to NaCl absorption to preserve extracellular fluid volume or drive renal K^+^ secretion to maintain K^+^ balance. First, we will discuss aspects of site-specific ENaC regulation in the context of Na^+^ homeostasis and blood pressure control. Subsequently, we will highlight implications of site-specific ENaC regulation for K^+^ homeostasis.

### ENaC activity in DCT2 and early CNT is critically involved in blood pressure control

As pointed out above, Liddle syndrome and PHA1 provide proof of concept that ENaC is a critical effector of long-term blood pressure control. Initial patch clamp studies demonstrated that ENaC activity in the CCD was readily observed only in animals treated with mineralocorticoid hormones or maintained on a low sodium diet [46–48, 110]. This led to the concept that renal ENaC activity is strictly aldosterone-dependent and probably essential in states of Na^+^ depletion but playing a minor role when dietary Na^+^ intake is normal or high. This paradigm was challenged when patch clamp studies in microdissected mouse tubules demonstrated that ENaC activity is aldosterone-independent in the transition zone from DCT2 to CNT (DCT2/CNT) [106, 113]. In this early part of the ASDN, sizeable ENaC currents were detected in mice maintained on a standard or even high Na^+^ diet and were shown to be preserved in aldosterone-deficient mice. Subsequent studies from different laboratories confirmed aldosterone-independent ENaC activity in DCT2/CNT [185, 190]. Moreover, it has recently been shown that ENaC activity in DCT2/CNT is aldosterone-independent but to a large extent MR-dependent [90, 104, 185]. Results from global and tissue-specific knockout (KO) mouse models also support the concept of site-specific roles of aldosterone and MR in controlling ENaC function in the ASDN. Importantly, global MR deficiency leads to a more severe renal phenotype than deficiency of aldosterone or deficiency of MR restricted to CD principal cells [13, 91, 132]. Similarly, CD-specific KO of the α-subunit of ENaC results in a relatively mild phenotype [146], comparable to the phenotype of MR KO in CD principal cells [132]. In contrast, global knockout of β-ENaC results in a severe salt-losing syndrome with hyperkalemia and neonatal death, which is similar to the phenotype caused by global MR KO [94].

Surprisingly, Liddle mice exhibited enhanced ENaC activity in CCD only when plasma aldosterone levels were high [33]. In contrast, patients with Liddle syndrome typically have suppressed plasma aldosterone levels. This raised the question where renal ENaC is hyperactive in Liddle syndrome to cause salt-sensitive hypertension. This question was answered by patch clamp studies demonstrating profound hyperactivity of ENaC in the DCT2/CNT from Liddle mice [107]. In particular, the failure of Liddle mice to suppress ENaC activity in DCT2/CNT when maintained on a high Na^+^ diet is consistent with the observation that Liddle mice develop hypertension under these conditions [126]. These findings indicate that appropriately adjusted ENaC activity in the DCT2/CNT is critically important for long-term blood pressure control. The high glucocorticoid and angiotensin II levels in aldosterone synthase–deficient mice with preserved ENaC activity in DCT2/CNT [106] suggest a role of these hormones in stimulating ENaC in this part of the ASDN. A stimulatory glucocorticoid effect is consistent with the findings that ENaC is aldosterone-independent but MR-dependent in DCT2/CNT [104], and that MR antagonists cause natriuresis in the absence of aldosterone [90]. In addition, angiotensin II stimulates ENaC in DCT2/CNT by an MR-independent mechanism [185], which underscores the likely importance of this nephron segment for upregulating Na^+^ absorption in states of volume depletion. It is tempting to speculate that pathophysiologically increased ENaC activity in the DCT2/CNT, possibly in combination with an increased aldosterone sensitivity of ENaC in the CNT/CCD [107], may not be limited to Liddle syndrome but may contribute to more common forms of salt-sensitive hypertension. Therefore, it will be an important task of future studies to elucidate the specific hormonal, local, and molecular factors involved in ENaC regulation in the DCT2/CNT.

### Aldosterone-independent ENaC activity in DCT2/CNT drives baseline K^+^ secretion

The concept of aldosterone-independent ENaC activity in DCT2/CNT has important implications for renal K^+^ secretion, because it implies that the electrical driving force generated by ENaC-mediated Na^+^ absorption and needed for K^+^ secretion does not depend on aldosterone in DCT2/CNT. Indeed, there is a need for aldosterone-independent ROMK-mediated K^+^ secretion, because the kidney maintains its ability to excrete K^+^ also when plasma aldosterone is low [161, 171, 190]. The finding that ENaC activity is aldosterone-dependent in CNT/CCD but aldosterone-independent in DCT2/CNT suggests that under baseline conditions with low plasma aldosterone, ROMK-mediated K^+^ secretion mainly occurs in the DCT2/CNT. As mentioned earlier, aldosterone-independent MR activation by glucocorticoids can occur when this is not prevented by the action of 11ßHSD2. Although there is some uncertainty regarding the precise level of 11ßHSD2 expression in different parts of the distal nephron, it appears to be lower in the proximal portion of the ASDN than in its distal portion [1, 20, 71, 104]. Thus, 11ßHSD2 expression may be sufficiently low in DCT2/CNT to allow circulating glucocorticoids to activate MR in this part of the nephron. Interestingly, the inhibitory effect of a high-salt diet on ENaC activity in the DCT2/CNT is minor compared with its large inhibitory effect in the CNT/CCD [106, 107]. This is consistent with the concept that a certain level of constitutive ENaC activity in the DCT2/CNT has to be maintained even in the context of high NaCl intake to preserve the kidney’s ability to secrete K^+^. The critical importance of ENaC activity in the DCT2/CNT for renal K^+^ secretion is also supported by studies using knockout mouse models. Mice with conditional knockout of ENaC in the CD were able to maintain K^+^ balance when challenged by high-K^+^ diet [146]. In contrast, mice with partial ENaC knockout in the CNT and possibly DCT2 developed hyperkalemia under similar experimental conditions [125]. Moreover, in aldosterone synthase–deficient mice placed on a high-K^+^ diet, high apical expression of ROMK was observed in the DCT2 and CNT but not in the CCD [171]. Confirming the functional importance of the DCT2/CNT for renal K^+^ secretion, recent patch clamp studies in microdissected tubules demonstrated that baseline ROMK activity is higher in DCT2/CNT than in CCD [105, 192]. The high baseline activity of ENaC and ROMK in the DCT2/CNT provides a regulatory potential for an adaptive inhibition of these channels in response to a decrease in dietary K^+^ intake. Indeed, ENaC activity in DCT2/CNT was shown to be strongly downregulated in mice maintained on a low K^+^ diet [105, 192]. Interestingly, a concomitant downregulation of ROMK currents was observed in one study [192] but was not confirmed in another study [105]. In animals maintained on a high-K^+^ diet, ENaC currents increased modestly in DCT2/CNT but strongly in CNT/CCD consistent with an increase in plasma aldosterone in response to the high-K^+^ diet and aldosterone-sensitive ENaC in CNT/CCD [192]. The findings outlined above highlight the important role of the DCT2/CNT in regulating renal K^+^ secretion in an aldosterone-independent manner mainly by adjusting ENaC activity through mechanisms that remain to be elucidated.

## Speculations on the mechanism of coordinated regulation of NCC and ENaC in controlling ECF volume and K^+^ excretion

Na^+^ transport in the distal nephron serves both ECF volume regulation and maintenance of plasm K^+^ concentration, and hence, the coordination of NCC and ENaC is critical [7]. A variety of mechanisms contribute to determining the relative activity of NCC and ENaC in the three subsegments shown in Fig. 1. The effects of aldosterone to stimulate ENaC are well established, and the role of interstitial [K^+^] in controlling NCC in DCT through WNK kinase activity is increasingly well supported [121, 168]. Since aldosterone does not likely have a direct effect on NCC [32, 166], interstitial [K^+^] can play a central role in coordinating NCC activity with that of ENaC. But what about versa? Are there factors other than aldosterone implicated in coordinating ENaC activation with NCC?

As discussed in the section  “The aldosterone-MR-SGK1 signaling module regulates ENaC”, recent data support the idea that mTORC2 plays an important role in maintaining renal ENaC activity [56, 60] and in mediating acute regulatory effects of renal interstitial [K^+^] on ENaC in the distal nephron [161] (Fig. 2). Moreover, the effects of mTORC2 are strongly modulated by WNK1 [161], consistent with a prior report that WNK1 stimulates SGK1 and ENaC, independently of its kinase activity [189]. Additional regulatory interactions of SGK1 and WNK kinases have also been identified [62], and hence, WNK kinases might play a role in coordinating NCC and ENaC responses to interstitial [K^+^]. Such effects may be particularly important in the DCT2/CNT where MR-dependent ENaC activation is preserved even in the absence of aldosterone and ROMK activity is high [90, 104–106, 187]. Other regulators such as angiotensin II may also be implicated in NCC-ENaC coordination [24], and influence whether aldosterone is natriferic or kaliuretic [156]. How these various hormonal and local effects, in particular changes in interstitial [K^+^], are fully integrated remains unclear. Thus, despite recent progress toward understanding the intertwined regulatory systems that control distal tubular Na^+^ transport in a site-specific manner, several controversies and unanswered questions exist, which are exciting topics of ongoing and future research in the field.

## Data Availability

All data described in this manuscript is published and available through PubMed or other open access sources.
